# 

**Published:** 1790

**Authors:** 


### LONDON

### MEDICAL JOURNAL.

VOLUME THE ELEVENTH,

FOR THE YEAR 1790.

Surgeon General's Office
LIBRARY.
63450
Washington, D.C.

LONDON:

PRINTED for the EDITOR,

And sold by JOSEPH JOHNSON, N°. 72,

ST. PAUL'S CHURCH YARD.

M,DCC,XC.

### CATALOGUE OF BOOKS.

1. THE Firſt Principles of Chemiſtry. By *William Nicholſon*. 8vo. *Robinſons*, London, 1790.

2. A deſcriptive Catalogue of upwards of eleven Hundred Species and Varieties of herbaceous or perennial Plants; divided into ſix Columns, exhibiting at one View the Names, Magnitude, Soil and Situation, Time of Flowering, Colour of the Flowers, and native Country of each Species. To which is added, a Liſt of hardy Ferns for the Decoration of northern Borders; and the moſt ornamental Annuals. By *John Graefer*, Botanic Gardener to the King of Naples. 8vo. *Smeeton*, London, 1789.

3. A new-diſcovered Fact, of a relative Nature, in the Venereal Poiſon. By *Jeſſe Foot*, Surgeon. 8vo. *Becket*, London, 1790.

4. Diſſertatio Inauguralis Medica de Glacie Medicamine; Auctore *Joanne Georgio Schroeder*, Riga-Livono. 4to. Gottingæ, 1789.

5. Diſſertatio Inauguralis Botanico-Medica de Aſtragalo Exſcapo *Linn.* Auctore *Henr. Chriſt. Gottl. Endter*, Bremenſi. 8vo. Gottingæ, 1789.

6. Diſſer-

6. Diſſertatio Medica de Signis Mortis diagnoſticis dubiis cauté admittendis et reprobandis ; Auctore *Joanne Chriſtiano Steinfeld*, Med. Doct. 4to. Jena, 1788.

7. *G. L. Hoffmanni* Opuſcula Latina medici Argumenti ſeparatim prius edita nunc vero in unum collecta typis recudi curavit et præfatus eſt *H. Chavet*. 8vo. Munſter, 1789.

8. Septima Diſſertatio Botanica, quatuordecim genera Monadelpha continens, 24 Tabulis accurate delineata. Auctore *Antonio Joſepho Cavanilles*, Hiſpano-Valentino, Scientiarum Upſalenſis Academiæ Socio, &c. 4to. Pariſiis, 1789.

9. Floræ Luſitanicæ et Braſilienſis Specimen ; et Epiſtolæ ab Eruditis viris *Carolo a Linné* & *Antonio de Haen* ad *Dominicum Vandelli* ſcriptæ. 4to. Conimbricæ, 1788. c. Tab. æneis v.

10. Hiſtoria præcipuorum Experimentorum circa Analyſin Chemicam Aeris atmoſphaerici uſumque principiorum ejus in componendis diverſis Naturæ corporibus. Auctore *Frid. Ludovico Schurer*, Argentinenſi. 4to. Argentorati, 1789.

